# Plant Growth Monitoring: Design, Fabrication, and Feasibility Assessment of Wearable Sensors Based on Fiber Bragg Gratings

**DOI:** 10.3390/s23010361

**Published:** 2022-12-29

**Authors:** Daniela Lo Presti, Joshua Di Tocco, Sara Cimini, Stefano Cinti, Carlo Massaroni, Rosaria D’Amato, Michele A. Caponero, Laura De Gara, Emiliano Schena

**Affiliations:** 1 Department of Engineering, Università Campus Bio-Medico di Roma, 00128 Rome, Italy; 2 Department of Science and Technology for Humans and the Environment, Università Campus Bio-Medico di Roma, 00128 Rome, Italy; 3 Department of Pharmacy, University of Naples Federico II, 80138 Naples, Italy; 4 Fusion and Technologies for Nuclear Safety and Security Department, FSN-TECFIS-MNF, ENEA Research Center of Frascati, 00044 Rome, Italy

**Keywords:** plant wearables, fiber optics, fiber Bragg gratings, metrological assessment, microenvironmental influence evaluation

## Abstract

Global climate change and exponential population growth pose a challenge to agricultural outputs. In this scenario, novel techniques have been proposed to improve plant growth and increase crop yields. Wearable sensors are emerging as promising tools for the non-invasive monitoring of plant physiological and microclimate parameters. Features of plant wearables, such as easy anchorage to different organs, compliance with natural surfaces, high flexibility, and biocompatibility, allow for the detection of growth without impacting the plant functions. This work proposed two wearable sensors based on fiber Bragg gratings (FBGs) within silicone matrices. The use of FBGs is motivated by their high sensitivity, multiplexing capacities, and chemical inertia. Firstly, we focused on the design and the fabrication of two plant wearables with different matrix shapes tailored to specific plant organs (i.e., tobacco stem and melon fruit). Then, we described the sensors’ metrological properties to investigate the sensitivity to strain and the influence of environmental factors, such as temperature and humidity, on the sensors’ performance. Finally, we performed experimental tests to preliminary assess the capability of the proposed sensors to monitor dimensional changes of plants in both laboratory and open field settings. The promising results will foster key actions to improve the use of this innovative technology in smart agriculture applications for increasing crop products quality, agricultural efficiency, and profits.

## 1. Introduction

Climate change and population growth are posing an increasing pressure on agricultural outputs. Hence, there is an urgent need to meet the demands imposed by these environmental and demographic issues for ensuring the sustainable growth of crop yields [1,2].

Plants supply food to nearly all terrestrial organisms, including humans. Hence, the growth monitoring of these living organs is important to understand the influence of environmental factors on crop productivity and to optimize plant development in a timely manner. Among others, microclimate, which refers to the climatic condition in the immediate nearby environment of plant organs, is critical because it regulates and affects the physiological reactions of the plants as well as the energy exchange activities between the plant and its surroundings. For instance, soil moisture and local temperature may affect plant water use efficiency and its waterlogging mechanisms. Both drought and waterlogging may cause a deficit in energy and mineral nutrient uptake, leading to a decline in plant growth and the nutritional value of its edible parts.

Nowadays, plant growth has been quantitatively assessed by measuring changes in organ dimensions in terms of length, height, or width of stem, leaves, and fruits or increases in the number of leaves and fruits. Until now, traditional methods for monitoring plant development involve remote sensing technologies such as spectroscopy, machine vision systems, and drones [3,4]. However, the lack of high spatial and temporal resolution associated with these methods makes them inadequate for the accurate monitoring of plant growth and the continuous tracking of plant organs development.

An ideal sensor for plant health monitoring should be easily anchored to different plant organs to profile various trait biomarkers (e.g., growth, water uptake, waterlogging tolerance, nutrients uptake, transport, etc.) and microenvironmental parameters such as temperature (T), relative humidity (RH), and light intensity. Moreover, it should be extremely stretchable and compliant with biological surfaces to cohabitate with plants without limiting any essential function. These characteristics can be found in the flexible sensors recently proposed for developing new wearable devices for humans. Distinctive features of these systems include high softness for better compliance with the human body, lightweight, unobtrusive design, and improved user conformability [5,6,7,8,9]. Although these wearable systems are already impacting healthcare by enabling the continuous and real-time monitoring of human health, their use in agricultural settings is still significantly left behind [10,11,12,13,14]. A few studies have explored the use of wearables for monitoring plant growth. These systems mainly consist of electrical sensors directly brushed on the plant surface [15,16] or encapsulated within stretchable matrices before their placement on plant organs [17,18]. However, some issues encountered by this technology (e.g., the negative influence of microclimate changes on the sensor performances, limited power supply, and data storage capacities) are still dampening their widespread uses in the smart farming [13,19,20]. Recently, a few studies have paved the way for the use of fiber optic sensors (i.e., fiber Bragg gratings—FBGs) in the monitoring of plant growth parameters by exploiting the FBGs’ high sensitivity to strain (ε), their multiplexing capacity, their high signal reliability, their miniaturized size, and light weight [21]. These properties may considerably simplify the sensor encapsulation process into a flexible substrate, improve the sensor density within the wearable system with reduced encumbrance and weight, and make the proposed solutions more robust and adaptable to curvilinear surfaces than not-encapsulated FBG sensors. Nowadays, wearables based on FBG technology have been mainly applied to human health monitoring where compliance with the body and a skin-like appearance are fundamental requirements for improving the system’s adaptability and conformability to the body [9,22].

In recent years, the application of this technology to smart agriculture aimed at overcoming some of the issues the electrical sensors face in this scenario. In recent studies, FBG-based plant wearables were developed for measuring stem elongation and environmental parameters [21]. In 2021, a multi-sensory platform consisting of a dumbbell-shaped flexible sensor was used for monitoring the growth of a tomato plant combined with two microenvironmental sensors for T and RH measurements [21]. Tests were carried out in indoor and outdoor scenarios for approximately ~20 h with interesting results. One year later, we applied a similar platform to a tobacco plant to monitor its growth [23]. Acquisitions lasted ~40 h and confirmed the capability of the proposed measuring system to monitor stem elongation. Hence, even in the case of different stem sizes and growth rates, the proposed system showed high performance in the growth monitoring [21,23].

In the present study, we extended our previous work [23] by proposing a further description and performance analysis of the dumbbell-shaped sensor and by introducing the first FBG-based plant wearable sensor for monitoring fruit circumferential expansion. Here, two different designs were proposed according to the plant organ on which the sensors are intended to be placed: a dumbbell for the stem and a ring for the fruit. Then, we investigated the sensing elements’ response to ε and the influence of two environmental parameters (T and RH) on the sensors’ performance. Finally, we detailed the tests carried out to assess the capability of the proposed wearable sensors to monitor plant growth in different scenarios.

## 2. Plant Wearables for Growth Monitoring: Design, Fabrication, and Metrological Characterization

This section focuses on the plant wearables proposed for growth monitoring from design to metrological characterization. We describe the chosen designs and the sensors’ working principle. Then, we present the fabrication process followed by tests and results of the metrological characterization carried out for investigating the FBGs’ sensitivity once encapsulated into a flexible substrate. Finally, the influence of T and RH on the plant wearables’ performance was evaluated.

### 2.1. Background and Working Principle of FBG Sensor

**Figure 1:**
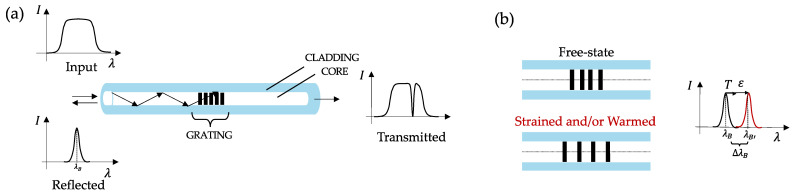
FBG working principle (**a**) and changes in its structure due to axial ε and T increments (**b**).

An FBG is a distributed Bragg reflector formed within the core of a short segment of an optical fiber by introducing a periodic variation in its refractive index [24]. An FBG reflects back specific wavelengths of propagating light generated by a broad-spectrum interrogation source while the others proceed through (see Figure 1a). The central wavelength of the reflected spectrum is the so-called Bragg wavelength (λ_B_) and satisfies Bragg’s condition:

(1) $$\lambda_{B}=2{\;n}_{\mathrm{eff}}\Lambda$$

where $n_{\mathrm{eff}}$ is the effective refractive index of the fiber core and Λ is the grating period. When the FBG is strained or warmed, a shift of $\lambda_{B}$ occurs (Figure 1b) given by:

(2) $$\frac{{\Delta \lambda }_{B}}{\lambda_{B}{}_{}}=K_{\varepsilon }\varepsilon +{\;K}_{T}T\;$$

The first term is related to the effect of ε on the grating and the $K_{\varepsilon }$ coefficient is the ε sensitivity depending on the physical elongation of Λ and the strain–optic coefficient of the fiber. The second term represents the effects of T on the grating with the $K_{T}$ coefficient and the T sensitivity, depending on the thermal expansion and the thermal–optic coefficients of the fiber. 

### 2.2. The Development of Wearables Based on FBG Technology for Plant Growth Monitoring

#### 2.2.1. Design

**Figure 2:**
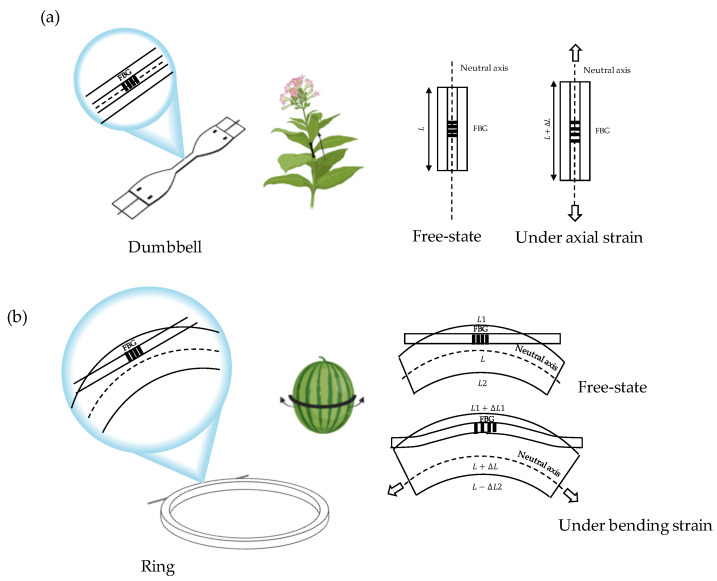
The two shapes of the proposed plant wearables for in-length (**a**) and in-width (**b**) growth monitoring.

In this study, we proposed two FBG-based sensors for growth monitoring. The sensors’ design was chosen according to the plant organ characteristics and growth dynamics (see Figure 2).

In summary, an FBG is sensitive to ε, especially the one applied along the fiber longitudinal axis. Hence, we shaped the sensor for stem elongation monitoring as a dumbbell. The dumbbell (Figure 2a) was designed to make the encapsulated FBG more responsive to longitudinal elongations. Such a shape enables the concentration of ε in the narrower part where the FBG is located, leading to an increment of λ_B_ when strained (that means that the engineered stem is elongating longitudinally). For circumferential growth monitoring, we proposed a ring-shaped sensor with a partial fiber optic encapsulation (i.e., its sensing part). In more detail, only the FBG sensor was integrated into the ring-shaped polymer matrix with an offset position from the neutral axis as depicted in Figure 2b. The chosen sensor positioning within the matrix guarantees a positive bending of the optical fiber and, in turn, an increment of λ_B_ when the radius increases (that means that the engineered fruit is expanding circumferentially). Moreover, a reduced encapsulation length prevents fiber breakage under strain when melon experiences dimensional changes due to growth.

#### 2.2.2. Fabrication Process

**Figure 3:**
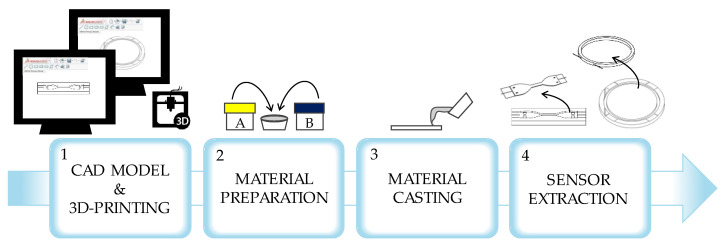
Main steps of the fabrication process.

Both the sensors were fabricated by following the same process consisting of four main steps as shown in Figure 3:

1. *The development of a 3D computer model of the mold.* We used a CAD software (Solidworks 2021) to design a mold with a negative impression for shaping the casting material and making the intended shapes.
2. *The preparation of the casting material*. We mixed the two parts (part A and part B) of a liquid bicomponent silicone rubber (i.e., Dragon Skin^TM^ 20, Smooth-On, Macungie, PA, USA) in an equal ratio by weight (1A:1B). Then, the mixture was degassed to remove the air bubbles.
3. *The casting of the material*. We positioned the optical fiber inside the mold in a pre-tensioned state. Then, we delivered the silicone into the mold.
4. *The curing of the silicone*. After 4 h, the silicone matrix was cured, and the sensor was extracted from the mold, peeling away any excess of silicone.

The dimensions of the dumbbell-shaped sensor are 45 mm (length) × 6 mm (width) × 1 mm (thickness), and the ones of the ring-shaped sensor are 55 mm (diameters) × 3 mm (width).

#### 2.2.3. Metrological Characterization

The FBG encapsulation into a polymer structure determines changes in response to ε and T compared to an unencapsulated sensor since the structural and thermomechanical properties of the material used to embed the grating may affect the transduction mechanism of the physical signals applied to the matrix surface into ${\Delta \lambda }_{B}$ of the encapsulated FBG sensor. It is worth noting that the response of a not-encapsulated FBG to ε is expected to be almost linear as well as the response to T changes (ΔT). However, the presence of a stretchable polymer matrix used to embed the grating may induce changes in the linearity of the FBG behavior and, consequently, in the $K_{\varepsilon }$ and $K_{T}$ values. Moreover, the polymer may also experience dimensional changes due to RH according to its level of hygroscopic behavior. Hence, we also investigated the influence of RH on the response of the two proposed plant wearable sensors.

#### Response to Strain of the Plant Wearable Sensors

The $K_{\varepsilon }$ investigation was performed using a different setup according to the sensor shape. The $K_{\varepsilon }$ value of the dumbbell-shape sensor was investigated by gripping its edges to the grips of a tensile testing for applying quasi-static axial ε values. The initial length (l_0_) of the sensors between the upper and the lower grid of the machine was 12 mm and the applied ε ranged from ~0% up to 2% of l_0_ at 1 mm·min^−1^ and room T. The applied input values were recorded by the Bluehill software, while the output of the FBG was encapsulated within the dumbbell by an FBG interrogator (si255 HYPERION platform, LUNA Inc., Roanoke, VI, USA). In both cases, a value of 100 Hz was set as the sampling rate. 

Data were analyzed in MATLAB environmental to obtain the calibration curve $\Delta \lambda_{B}$ vs. ε and the $K_{\varepsilon }$ value (for further details see [23]).

**Figure 4:**
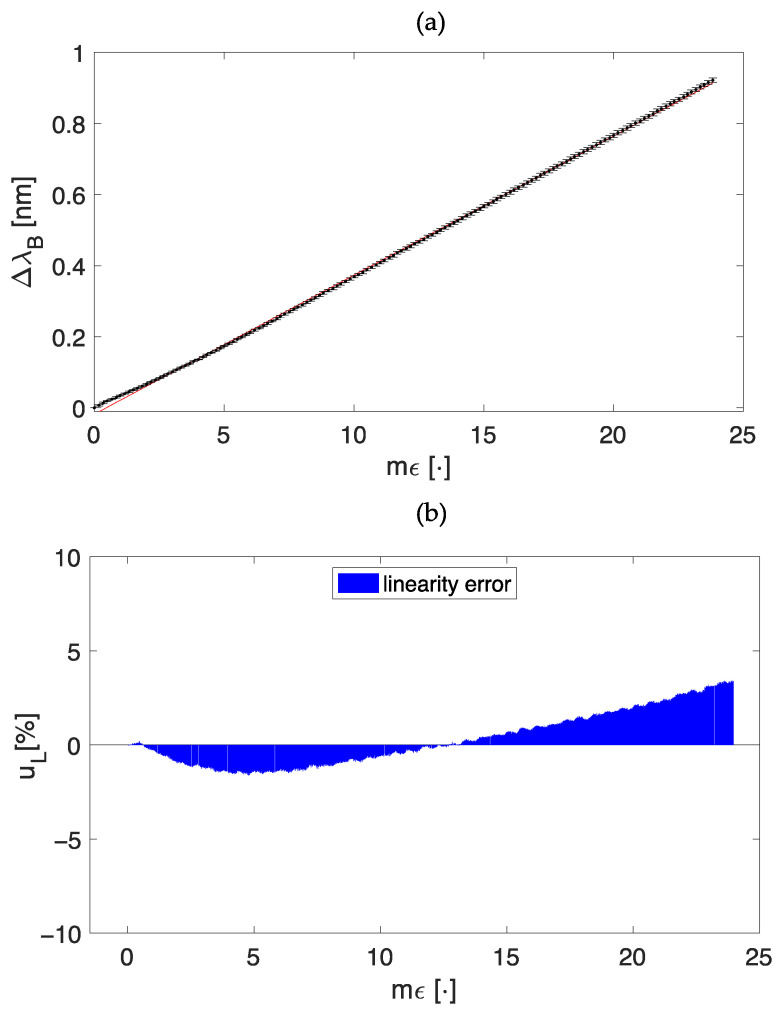
The calibration curve $\lambda_{B}$ vs. mε (**a**), and the bar plot of the linearity error u_L_ (**b**).

Figure 4a shows the response of dumbbell-shaped plant wearable sensor to the applied ε in terms of mean $\Delta \lambda_{B}$ values and uncertainty together with the best fitting curve. Despite the use of a polymer encapsulation, the FBG sensor within the dumbbell-shaped flexible matrix maintained a linear response with a $K_{\varepsilon }$ value of 0.04 nm·mε^−1^.

The high agreement between the experimental data and the model is testified by the high value of R^2^ (i.e., >0.99). Moreover, to better quantify this linear behavior, we evaluated the linearity error ($u_{L}$) defined as:

(3) $$u_{L}\left[\%\right]=\;100\;\cdot \;(\Delta \lambda_{B}{\left(\varepsilon \right)}^{\exp}-\Delta \lambda_{B}{\left(\varepsilon \right)}^{\mathrm{th}})/\Delta \lambda_{B}{\left(\varepsilon \right)}^{\exp^{\mathrm{fs}}}\;$$

where $\Delta \lambda_{B}{\left(\varepsilon \right)}^{\exp}$ is the experimental value of $\Delta \lambda_{B}$ at a specific ε, $\Delta \lambda_{B}{\left(\varepsilon \right)}^{\mathrm{th}}$ is the theoretical value of $\Delta \lambda_{B}$ at the same ε obtained by the linear model, and $\Delta \lambda_{B}{\left(\varepsilon \right)}^{\exp^{\mathrm{fs}}}$ is the full-scale $\Delta \lambda_{B}$ range. The maximum $u_{L}$ value for the dumbbell shape is 3.40% (see Figure 4b).

**Figure 5:**
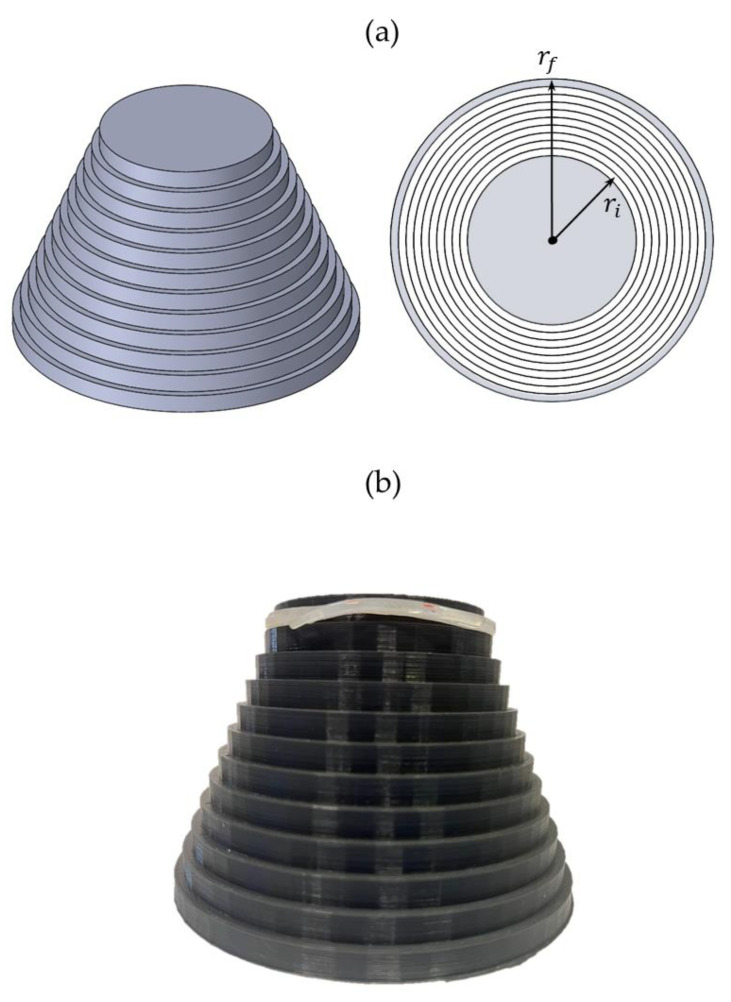
Design of the ring tower (**a**), the printed structure with the ring-shaped sensor placed around the first ring (**b**).

The ring-shaped plant wearable sensor response to ε when subjected to bending was investigated by applying different curvature radii to the matrix. To perform this test, a ring-tower tool was designed and printed in polylactic acid (PLA). It consists of 11 concentric rings with different radius values ranging from 27.5 mm ($r_{i}$) to 52.5 mm ($r_{f}$) at steps of 2.5 mm as shown in Figure 5a. The overall height of the structure is 77 mm (7 mm of height for each ring enclosed within the final tower structure). The static calibration of the ring-shaped sensor was carried out as follows. The ring-shaped sensor was moved from the first to the last ring (see Figure 5b) to apply known curvatures to the sensing element. Each curvature was kept by the sensor for approximately 10 min before moving to the following circumference. Each curvature is defined by a specific bending radius (from $r_{i}$ to $r_{f})$. Changes in the FBG output within the matrix were recorded by an FBG interrogator (si255 HYPERION platform, LUNA Inc.) at a sampling frequency of 100 Hz. The $\Delta \lambda_{B}$ changes experienced by the ring-shaped sensor can be considered mainly caused by the ε varying accordingly to the applied bending radius. 

Data were analyzed in MATLAB environment as follows. The Δλ_B_ values recorded over the 10 min in which the same bending radius was maintained were averaged to obtain the mean Δλ

**Figure 6:**
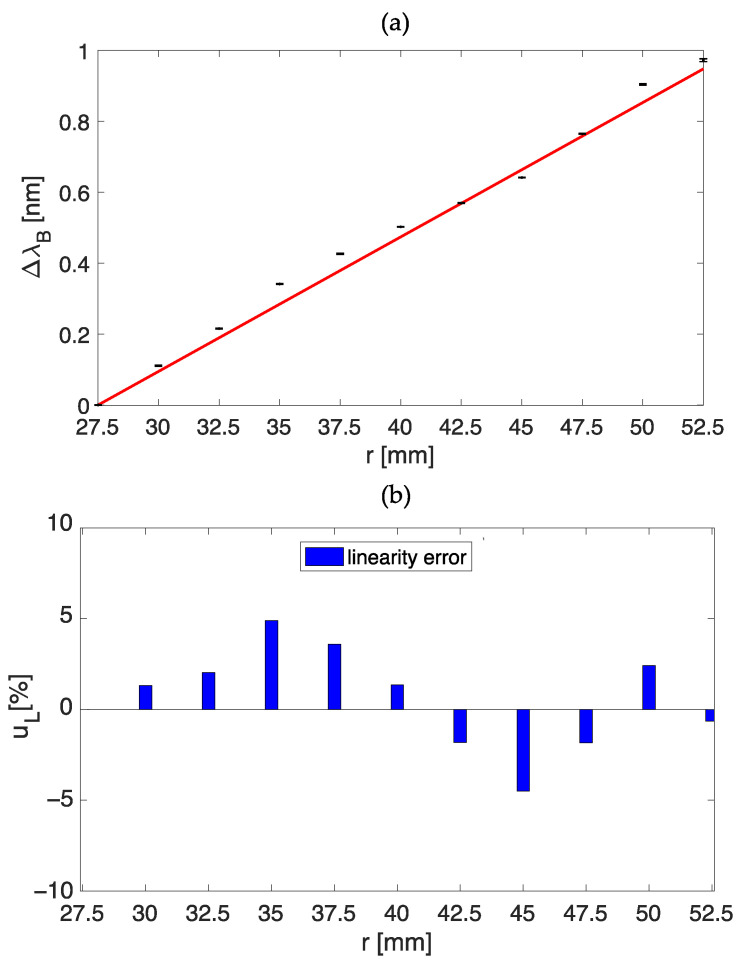
The calibration curve $\lambda_{B}$ vs. r (**a**), and the bar plot of the linearity error $u_{L}$ (**b**).

_B_ value corresponding to the applied curvature. Then, the standard deviation was also computed for each 10-min lasting step. The calibration curve Δλ_B_ vs. Δr is shown in Figure 6a. The experimental data follow a linear trend. The high agreement between the experimental data and the model is testified by the high value of R^2^ (i.e., >0.99) and, according to Equation (3), a value of $u_{L\;}$ = 4.9 % (Figure 6b). The slope of the best fitting line can be used as an estimation of the ring-shaped sensor sensitivity to bending ε ($S_{\varepsilon }$), with a value of $S_{\varepsilon }$ = 0.04 nm/mm.

#### Response to Temperature of the Dumbbell- and Ring-Shaped Plant Wearable Sensors

**Figure 7:**
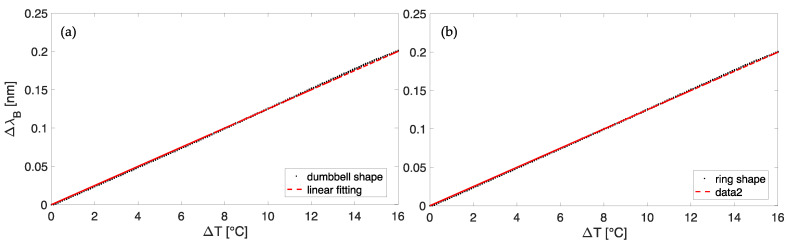
The calibration curve $\lambda_{B}$ vs. ΔT of the sensor with a dumbbell shape (**a**) and a ring shape (**b**).

The $K_{T}$ investigation was performed using a laboratory oven to expose the two wearable sensors to the same T measuring range (i.e., from T^min^ of 22 °C to T^max^ of 38 °C). A thermistor was placed within the oven close to the sensors to measure reference T values. The static calibration was carried out as follows. Once T^max^ is reached, we turned off the oven and allowed it to cool down to ambient T. Data from the wearables and the reference probe were recorded at a sampling rate of 1 Hz and analyzed in MATLAB environment to obtain the calibration curve $\lambda_{B}$ vs. ΔT. The linear trends in Figure 7a,b with a slope of ~0.01 nm/°C (i.e., $K_{T}$) for both the plant wearable sensors confirmed no considerable influence of the polymer matrix on the encapsulated FBG response to T. Indeed, the estimated values of K_T_ are comparable to the one of a not-encapsulated FBG sensor.

#### Response to Relative Humidity of the Dumbbell- and Ring-Shaped Plant Wearable Sensors

We expected a negligible influence of RH on the output of the encapsulated FBG-based sensor since both the FBGs and the material used for encapsulating the gratings are not hygroscopic; hence, no additional strains on the FBGs’ output should occur. However, considering potential applications of the proposed plant wearable sensors in outside settings under uncontrolled environmental conditions, the influence of RH changes on the FBG response was carefully assessed. Both the proposed plant wearable sensors were placed within a custom climatic chamber and exposed to quasi-static RH variations. Humified air was forced into the chamber to reach a level of RH of ~90%. Then, dry air was forced inside the chamber at a flow rate of 1 L·min^−1^ to lower the RH to ~20%. Reference RH values were recorded by a capacitive-based RH sensor (HIH 4000-002, Honeywell International Inc., Morristown, NJ, USA, accuracy = ±3%) connected to a data acquisition board (NI DAQ USB-6009, NI Instruments, Austin, TX, USA) that record RH changes at a sampling rate of 100 Hz.

**Figure 8:**
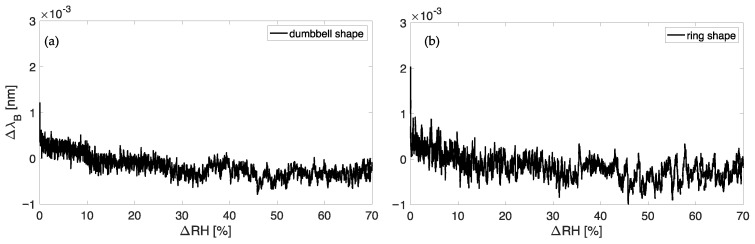
The calibration curve $\lambda_{B}$ vs. ΔRH of the sensor with the dumbbell shape (**a**) and with the ring shape (**b**).

The same value was set on the FBG interrogator used to record the plant wearable Δλ_B_ values during the test_._ Data were analyzed in MATLAB environment to obtain the calibration curves $\lambda_{B}$ vs. ΔRH for the dumbbell-shaped (Figure 8a) and ring-shaped (Figure 8b) sensors.

As shown in Figure 8a,b, negligible changes were found in the plant wearable response to RH over a wide measurement interval. This result suggests that the effect of such an environmental quantity on the output of the proposed sensors may be considered negligible even under challenging environmental conditions. Therefore, conversely from T, no compensation of RH is needed on the output of the FBGs within the polymer matrices.

## 3. Plant Wearables for Growth Monitoring: Test, Data Analysis and Results

### 3.1. Tests to Assess the Capability of Wearables Based on FBG Technology for Plant Growth Monitoring

The capability of the proposed wearable sensors for monitoring stem and fruit growth was assessed in laboratory and in-field settings. A dummy sensor (i.e., an unencapsulated FBG sensor) in unstrained conditions was used in both the scenarios to compensate for the influence of T on the output of the FBG sensors encapsulated within the dumbbell- and ring-shaped matrices.

#### 3.1.1. Dumbbell-Shaped Plant Wearable Sensor Assessment

**Figure 9:**
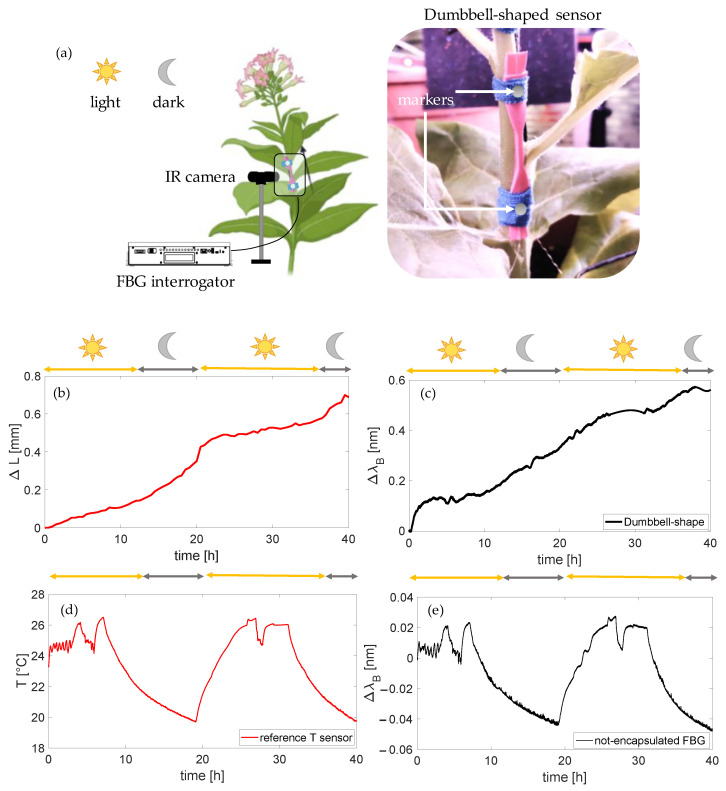
The setup used for in-laboratory tests including the FBG interrogator, the IR camera, and the platform for reference T measurements with a picture of the dumbbell-shaped sensor around the stem with tapes at the matrix ends and markers for reference elongation measurements (**a**); reference elongation trend over time (**b**); dumbbell-shaped sensor output changes after T compensation (**c**); reference T sensors output changes over time (**d**); and not-encapsulated FBG sensor output changes over time (**e**).

The capability of the dumbbell-shaped sensor to monitor stem growth was assessed on a tobacco plant as already described in [23]. A similar system was also proposed in [21] to monitor the stem elongation of a tomato plant, suggesting a good adaptability of the proposed shape to different stem widths (see Figure 9a,b).

Tobacco (Nicotiana tabacum) seeds were sterilized in 70% ethanol for 2 min and treated with 5% bleach with a soaking time of 15 min. Then, seeds were rinsed with sterilized water five times and sowed in the soil at 25 ± 1 °C with a photoperiod of 16 h light/8 h dark in a laboratory setting.

The dumbbell-shaped plant wearable sensor was installed on the stem of the tobacco plant after two months of seed germination by using a biocompatible tape, as depicted in Figure 8a. The laboratory settings allowed for an easy installation of two 6 mm diameter photo-reflective markers (3M^TM^ Schotchlite^TM^ Reflective Material 8910 Silver Fabric, SF Vest location, Puidoux, Switzerland) at the extremity of the dumbbell-shaped sensor and the use of an IR camera (Longruner LC26-MakerBot, New York, NY, USA, image resolution 2592 × 1944 pixels) without any external disturbance such as wind or wild animals. The IR camera was used to capture photos for spatially tracking changes in the marker distance and obtain reference elongation values (ΔL). Finally, a Raspberry Pi4 was used as power source for the IR camera and for high-resolution images recording every 5 min using Python. The dummy sensor was multiplexed to the flexible sensor for enabling the T monitoring and compensating the $\Delta \lambda_{B}$ values induced by thermal effects. In fact, literature studies showed that the thermal sensitivity of an FBG encapsulated within a Dragon-skin silicone matrix is comparable to the one of an unencapsulated sensor [7,8]; hence the $\Delta \lambda_{B}$ values experienced by the dummy sensor may be subtracted to the ones of the dumbbell-shaped sensor to perform the T compensation. To better analyze the thermal influence, reference values of T were measured by using a commercial system (BME280 by BOSCH) with a sampling rate of 10 Hz. Data acquisition lasted approximately 40 h. The same module was used to monitor reference RH values and a chitosan-based FBG sensor was used for the same scope since both non-encapsulated and silicone-encapsulated sensors have the potential to detect environmental changes in terms of RH. Further information about these results have been proposed in [23].

Data were analyzed in MATLAB environment. Figure 9b,d illustrate the reference marker distance (ΔL) trend and the environmental T trend over time, showing a stem growth rate of ~0.017 mm/h and a ΔT of ~6.8 °C between the two light–night cycles. Figure 8c,e show the output changes of the dumbbell-shaped sensor used for monitoring stem elongation after T compensation and the ones of the non-encapsulated FBG sensor. The dumbbell-shaped sensor showed promising capacities in monitoring stem growth with a sensitivity of 0.82 nm·mm^−1^ (see Figure 9b,c). Moreover, the non-encapsulated sensor, as expected, followed the trend of the reference T sensor (see Figure 9d,e).

#### 3.1.2. Ring-Shaped Plant Wearable Sensor Assessment

**Figure 10:**
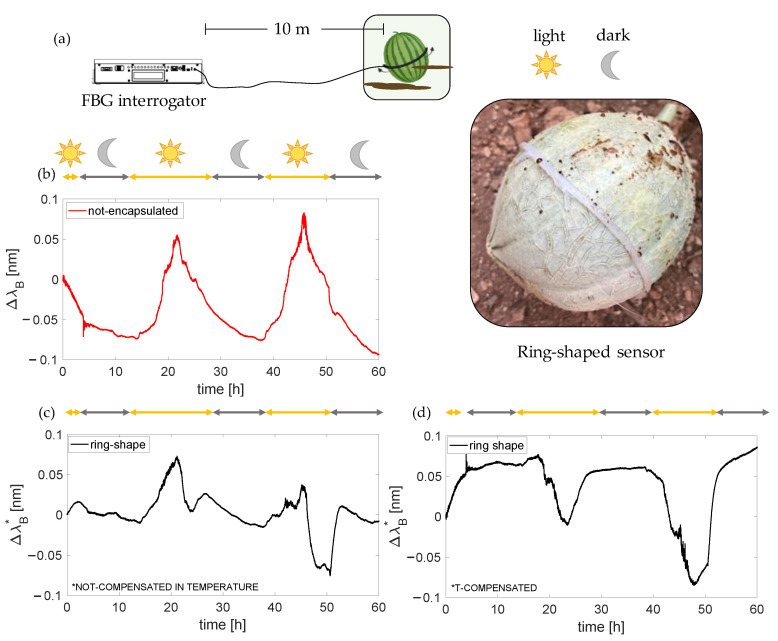
The setup used for in-field tests including the FBG interrogator with a picture of the ring-shaped sensor around the fruit (**a**); the non-encapsulated FBG sensor output changes over time (**b**); the ring-shaped sensor output changes before (**c**) and after T compensation (**d**).

The capability of monitoring fruit growth was assessed in an in-field scenario by placing the proposed ring-shaped plant wearable sensor around a cantaloupe fruit (Cucumis melo), as shown in Figure 10a. Cantaloupe has an extensive shallow root system; hence, the plant must receive frequent watering for healthy growth and an adequate adaptation to the changing climate. The test in the open field lasted ~60 h, leading the sensor to be exposed to a dynamic environment with significant changes in terms of T, RH, and illumination. The plant was watered twice (i.e., when the acquisition started and after ~48 h). The influence of RH can be considered negligible since the output changes of both the non-encapsulated and silicone-encapsulated sensors are not affected by RH, as shown in Section 2. A dummy sensor (i.e., a non-encapsulated FBG) was used to measure microclimate T values and compensate the influence of T on the output of the ring-shaped sensor. The challenging scenario did not allow us to install the IR camera for recording reference measurements of growth. However, we performed two measurements of the fruit diameter, the first (i.e., 30.8 mm) after the sensor installation before starting the acquisition and the latter at the end of the 60 h lasting acquisition (i.e., 30.8 mm). Figure 10b shows the output of the non-encapsulated FBG sensor over time. As expected, the dummy sensor output change experienced an increase in $\Delta \lambda_{B}$ with T (see Figure 10a), reaching the maximum value when the sunlight directly hit the fruit for every night–day cycle. Figure 10c shows the raw data of the ring-shaped sensor, and Figure 10d shows the T-compensated one. As illustrated in Figure 10d, the fruit showed a dynamic circumferential change presumably induced by both growth and waterlogging mechanisms. In more detail, especially when the sun directly hit the fruit, a considerable reduction in $\Delta \lambda_{B}$ starts (this trend is visible during every night–light cycle). The first two cycles showed a similar $\Delta \lambda_{B}$ excursion between night and day. A possible explanation can be found in the fruit dehydration experienced with T, resulting in a dimension reduction due to water uptake depression. These results suggest no considerable changes in dimensions except for those due to water logging mechanisms. When the soil was watering (between the second and the third day), a rapid change in $\Delta \lambda_{B}$ is visible in Figure 10d. It is presumably related to fruit expansion due to water uptake from the soil. However, conversely to what happened during the first two cycles, $\Delta \lambda_{B}$ reached values higher than the ones reached in the first and the second night–light cycles, suggesting that together with the water logging mechanism (displayed in the rapid up and down trends for each cycle), a change in growth was experienced by the melon. These preliminary results show the capability of the proposed ring-shaped sensor to detect circumferential changes due to both waterlogging mechanisms and growth in an open-field scenario.

## 4. Discussions and Conclusions

This study proposed the use of FBG sensors for developing wearables to monitor plant growth. We focused on two specific plant organs: stem and fruit. According to the chosen organ, a different shape was conferred to the plant wearable sensor to improve its adaptability to the plant surface and its performance in detecting growth parameters. We proposed a dumbbell-shaped sensor axially positioned along the stem for elongation monitoring. For the detection of the circumferential expansion of the fruit, we developed a ring-shaped sensor. Metrological characterization and feasibility assessment of the sensors’ capability for working in both laboratory and open-field scenarios were carried out with promising results (see Section 3). Findings showed that both the sensors have high sensitivity to the inputs such as ε (0.04 nm·mε^−1^) and r (0.04 nm·mm^−1^) changes with a slight influence of T (0.01 nm·°C^−1^) and negligible effect of RH. In the literature, recent studies proposed using plant wearables to monitor plant organs’ growth (i.e., stem, leaves, and fruits). Most works used electrical sensors encapsulated within flexible substrates or directly printed on the plant surface. For instance, Nassar and coauthors proposed a stretchable dumbbell-shaped sensor based on the wrinkled gold metal wire within a polymer matrix made of Polydimethylsiloxane (PDMS) for monitoring stem elongation [18]. The sensor worked for 48 h, monitoring a growth of 905 μm per day. Tang and coauthors proposed the first study focusing on a plant wearable sensor fabricated for fruit growth monitoring [15]. A CH-based graphene ink was directly brushed on two cucumbers, and its output changes were monitored for 18 min. Another plant wearable sensor for monitoring fruit growth has been recently proposed [16]. As in the present study, the sensor has a ring shape to be placed around a pepper fruit. For 18 days, the resistance changes induced by the expansion of pepper fruits on the sensor were measured when the fruit was exposed and nonexposed to LED treatments in a controlled environment. The results show great sensor capacity for long-term uses and reveal a significantly higher growth rate in fruits exposed to LED treatments. In the last year, FBG sensors have been used for plant wearables’ development. To the best of our knowledge, we were the first to exploit the FBG advantages in smart farming and precision agriculture. We proposed FBG-based dumbbell-shaped sensors to measure the elongation of the stem of two different plants (i.e., a tomato plant and a tobacco plant) in controlled and uncontrolled settings.

Although some tests have been carried out in open-field scenarios, most state-of-the-art solutions have been proposed for short-term measurements in a controlled environment (e.g., laboratory settings, greenhouses, and growth chambers), and tapes were used to secure the sensor to the plant. Unfortunately, the plant transpiration and environmental changes may harden the sticky component, making the tape ineffective, but no further investigation about the system capability to exhibit a stable bio-interface adaptability and compatibility over longer acquisitions has been carried out yet. The direct brushing modalities (as proposed in [15]) may overcome these issues but requires nontoxic inks to cohabitate with plant organs without affecting their development. Even in this case, the influence of inks on the plant physiological processes has not been systematically assessed. A novel strategy to improve the system compliance with the plant surface has been proposed by using ring-shaped solutions for growth monitoring [16] as in the present study. In this way, no tapes are required, and no ink is brushed on the plant surface. However, these solutions have only been proposed for monitoring fruit expansion. No other growth parameters have been monitored by using a similar approach. Finally, most of the works presented in the literature have not considered the decoupling of the sensor output changes induced by plant physiological processes from the ones caused by microclimate variations. Here, we carried out some tests to assess the influence of environmental T and RH, and we proposed a method to compensate their influence by using a dummy sensor multiplexed to the developed plant wearables. In this way, the system performance was optimized even when the plant wearables worked in uncontrolled environments. Deeper investigations should be carried out to make the impact of environments negligible for improving the growth sensing performance of plant wearables.

In conclusion, this study proposes different sensors design tailored to plant organs. However, some issues must be addressed to boost their use in an open field over longer acquisition. One regards the anchorage modality of the dumbbell-shaped sensor for stem elongation monitoring since an additional tape must be used for securing the sensor ends to the plant surface. Further investigations will be carried out to better assess any hardiness of the sticky components, which may affect the tape effectiveness over long-lasting acquisitions and, therefore, the sensor performance. Regarding the sensor for fruit growth monitoring, the ring shape stabilizes the sensor adaptability to the wrinkled surface of the fruit in open-field scenarios. Future tests will be devoted to better assessing the sensors’ performance over longer acquisitions and evaluating the influence of challenging microenvironmental conditions and waterlogging mechanisms on the plant development.

We envision that fully integrated systems that capture plant health metrics according to the influence of biotic and abiotic factors will bring a new level of innovation in crop production by enabling actions that can be taken to improve the crop products’ quality, agricultural efficiency, and profits.

## Data Availability

Data available on request.

## References

[1] United Nations Department of Economics and Social Affairs (2019). World Population Prospects 2019: Highlights|Multimedia Library—United Nations Department of Economic and Social Affairs.

[2] Khreis H. (2020). Traffic, Air Pollution, and Health. Advances in Transportation and Health: Tools, Technologies, Policies, and Developments.

[3] Kanning M., Kühling I., Trautz D., Jarmer T. (2018). High-Resolution UAV-Based Hyperspectral Imagery for LAI and Chlorophyll Estimations from Wheat for Yield Prediction. Remote Sens..

[4] Mozgeris G., Jonikavičius D., Jovarauskas D., Zinkevičius R., Petkevičius S., Steponavičius D. (2018). Imaging from Manned Ultra-Light and Unmanned Aerial Vehicles for Estimating Properties of Spring Wheat. Precis. Agric..

[5] Pan J., Zhang Z., Jiang C., Zhang L., Tong L. (2020). A Multifunctional Skin-like Wearable Optical Sensor Based on an Optical Micro-/Nanofibre. Nanoscale.

[6] Hong Y.J., Jeong H., Cho K.W., Lu N., Kim D.H. (2019). Wearable and Implantable Devices for Cardiovascular Healthcare: From Monitoring to Therapy Based on Flexible and Stretchable Electronics. Adv. Funct. Mater..

[7] Lo Presti D., Massaroni C., D’Abbraccio J., Massari L., Caponero M., Longo U.G., Formica D., Oddo C.M., Schena E. (2019). Wearable System Based on Flexible Fbg for Respiratory and Cardiac Monitoring. IEEE Sens. J..

[8] Lo Presti D., Bianchi D., Massaroni C., Gizzi A., Schena E. (2022). A Soft and Skin-Interfaced Smart Patch Based on Fiber Optics for Cardiorespiratory Monitoring. Biosensors.

[9] Lo Presti D., Massaroni C., Jorge Leitao C.S., De Fatima Domingues M., Sypabekova M., Barrera D., Floris I., Massari L., Oddo C.M., Sales S. (2020). Fiber Bragg Gratings for Medical Applications and Future Challenges: A Review. IEEE Access.

[10] Lee G., Wei Q., Zhu Y. (2021). Emerging Wearable Sensors for Plant Health Monitoring. Adv. Funct. Mater..

[11] Galatus R.M., Papara R., Buzura L., Roman A., Ursu T. Wearable Multi-Sensor for Plant Monitoring, Based on Fluorescent Fibers. Proceedings of the SPIE.

[12] Dufil G., Bernacka-Wojcik I., Armada-Moreira A., Stavrinidou E. (2022). Plant Bioelectronics and Biohybrids: The Growing Contribution of Organic Electronic and Carbon-Based Materials. Chem. Rev..

[13] Qu C.C., Sun X.Y., Sun W.X., Cao L.X., Wang X.Q., He Z.Z. (2021). Flexible Wearables for Plants. Small.

[14] Lo Presti D., Di Tocco J., Massaroni C., Cimini S., De Gara L., Singh S., Raucci A., Manganiello G., Woo S.L., Schena E. (2023). Current Understanding, Challenges and Perspective on Portable Systems Applied to Plant Monitoring and Precision Agriculture. Biosens. Bioelectron..

[15] Tang W., Yan T., Ping J., Wu J., Ying Y. (2017). Rapid Fabrication of Flexible and Stretchable Strain Sensor by Chitosan-Based Water Ink for Plants Growth Monitoring. Adv. Mater. Technol..

[16] Hsu H.H., Zhang X., Xu K., Wang Y., Wang Q., Luo G., Xing M., Zhong W. (2021). Self-Powered and Plant-Wearable Hydrogel as LED Power Supply and Sensor for Promoting and Monitoring Plant Growth in Smart Farming. Chem. Eng. J..

[17] Zhao Y., Gao S., Zhu J., Li J., Xu H., Xu K., Cheng H., Huang X. (2019). Multifunctional Stretchable Sensors for Continuous Monitoring of Long-Term Leaf Physiology and Microclimate. ACS Omega.

[18] Nassar J.M., Khan S.M., Villalva D.R., Nour M.M., Almuslem A.S., Hussain M.M. (2018). Compliant Plant Wearables for Localized Microclimate and Plant Growth Monitoring. npj Flex. Electron.

[19] Hetzroni A., Miles G.E., Engel B.A., Hammer P.A., Latin R.X. (1994). Machine Vision Monitoring of Plant Health. Adv. Sp. Res..

[20] Zhang Q., Ying Y., Ping J. (2022). Recent Advances in Plant Nanoscience. Adv. Sci..

[21] Presti D.L., Cimini S., Massaroni C., D’amato R., Caponero M.A., De Gara L., Schena E. (2021). Plant Wearable Sensors Based on FBG Technology for Growth and Microclimate Monitoring. Sensors.

[22] Lo Presti D., Massaroni C., Schena E. (2018). Optical Fiber Gratings for Humidity Measurements: A Review. IEEE Sens. J..

[23] Lo Presti D., Di Tocco J., Massaroni C., Cimini S., Cinti S., D’amato R., Caponero M.A., De Gara L., Schena E. (2022). Fiber Optic Plant Wearable Sensors for Growth and Microclimate Monitoring. Proceedings of the 2022 IEEE International Workshop on Metrology for Industry 4.0 and IoT, MetroInd 4.0 and IoT.

[24] Erdogan T. (1997). Fiber Grating Spectra. J. Light. Technol..

